# Effect of treatment of peripheral arterial disease on the onset of anaerobic exercise during cardiopulmonary exercise testing

**DOI:** 10.14814/phy2.14815

**Published:** 2021-04-04

**Authors:** Mohamed Barkat, Angela Key, Tamara Ali, Paul Walker, Nick Duffy, Jayne Snellgrove, Francesco Torella

**Affiliations:** ^1^ Liverpool Vascular and Endovascular Service Liverpool University Hospitals NHS Foundation Trust Liverpool UK; ^2^ Institute of Ageing and Chronic Disease University of Liverpool Liverpool UK; ^3^ Department of Respiratory Medicine Liverpool University Hospitals NHS Foundation Trust Liverpool UK; ^4^ Faculty of Health and Life Sciences University of Liverpool Liverpool UK; ^5^ School of Physical Sciences University of Liverpool Liverpool UK; ^6^ Liverpool Centre for Cardiovascular Science Liverpool UK

**Keywords:** aerobic threshold, atherosclerosis, skeletal muscle

## Abstract

**Objective:**

Cardiopulmonary exercise testing (CPET) is often used to assess pre‐operative fitness in elderly patients, in whom peripheral arterial disease (PAD) is highly prevalent, but may affect the results of CPET by early lactate release due to muscle ischemia. This study investigated the effect revascularization of PAD on oxygen delivery (VO_2_) during CPET.

**Method:**

We conducted a prospective cohort study of 30 patients, who underwent CPET before and after treatment of ilio‐femoral PAD. The primary outcome measure was difference in VO_2_ at the lactate threshold (LT) before and after revascularization. Secondary outcome measures were the relationship between change in VO_2_ at LT and peak exercise and change in ankle–brachial index (ABI) differential.

**Results:**

The study was approved by the North West‐Lancaster Research and Ethics committee (reference 15/NW/0801) and registered in clinicaltrial.gov (reference NCT02657278). As specified in the study protocol, 30 patients were recruited but only 20 (15 men), with a mean age of 62 years, completed pre‐ and post‐treatment CPETs. Twelve patients demonstrated an improvement in VO_2_ at LT after revascularization, but the difference did not achieve statistical significance (mean difference (95% CI) = 1.43 (−0.21 to 3.08) ml/kg/min; (*p* = 0.085). There was, however, a significant improvement in VO_2_, VE/CO_2_, workload and Borg breathlessness and leg fatigue score at peak exercise after revascularization. There was no significant correlation between change in VO_2_ at LT (*r* = −0.11, *p* = 0.65) or change in VO_2_ at peak and ABI differential (*r* = −0.14, *p* = 0.55).

**Conclusion:**

Revascularization of PAD led to significant improvement in multiple peak/maximal exercise parameters within a few weeks and without exercise training. We were unable to demonstrate a statistically significant improvement in VO_2_ at LT albeit in a majority of subjects this exceeded what we pre‐defined as clinically significant.

## Graphical abstract

The study suggests that the presence of peripheral artery disease may affect measured oxygen delivery during cardiopulmonary test. Clinical interpretation of this test may need to take into account the presence of occlusive arterial disease of the lower limbs if these results are confirmed.

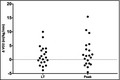

## 1 INTRODUCTION

Cardiopulmonary exercise testing (CPET) provides a safe, reliable, repeatable, non‐invasive, objective, individual assessment of combined pulmonary, cardiac, and circulatory function. It quantifies the functional ability to respond to the increased metabolic demands of major surgery generating a patient‐specific measure of risk (Moran et al., 2016). The evidence base supporting the value of preoperative CPET in identifying high‐risk patients undergoing major non‐cardiac surgery is increasing (Hightower et al., 2010; Older et al., 1999; Snowden et al., 2010; Wilson et al., 2010). CPET is also used as a preoperative risk‐stratification tool to predict postoperative mortality, length of stay (LOS), and morbidity; however, its role requires validation (Moran et al., 2016).

Anaerobic, or lactate, threshold (LT) marks the onset of anaerobic metabolism as a result of inadequate oxygen delivery and is not altered by patient effort. It is usually reached halfway through an incremental exercise test. In surgery, it has been found to predict postoperative morbidity and mortality, and can thus help triage the patient to an appropriate postoperative care facility.

Peripheral arterial disease (PAD) is highly prevalent in elderly patient affecting about 20% of people aged 60 years or older (Burns et al., 2003). Patients with PAD may develop ischemia during leg exercise not simply because of poor cardiorespiratory reserve and/or poor fitness, but because the blood supply to the leg muscles is impaired, resulting in early lactate release. Hence, oxygen uptake at lactate threshold (VO_2_ at LT) may poorly reflect cardiorespiratory status, risk and prognosis in PAD.

We hypothesized that, in patients with symptomatic ilio‐femoral PAD, impaired muscle blood flow would lead to reduced oxygen uptake at LT and that revascularization would improve VO_2_ in proportion to the improvement in peripheral circulation.

## 2 METHOD

### 2.1 Study design

The protocol of this study, which was approved by the North West‐Lancaster Research and Ethics committee (reference 15/NW/0801) and registered in clinicaltrial.gov (reference NCT02657278), has already been published (Key et al., 2016). In brief, consenting patients with ilio‐femoral occlusive PAD scheduled to undergo percutaneous or surgical revascularization underwent symptom‐limited CPET before and after surgical or endovascular correction of their PAD. Inclusion and exclusion criteria are summarized in Table 1.

**TABLE 1:** Entry criteria

Inclusion	Exclusion
Ability and willingness to give written informed consent	Critical ischemia as presenting symptom (rest pain and/or tissue loss)
Ilio‐femoral PAD scheduled for surgical or percutaneous treatment	Age <18 years
Ability to perform a CPET on a cycle ergometer	Previous amputation
Intermittent claudication as presenting symptom	Inability to perform a CPET on a cycle ergometer
Age ≥18 years	Uncontrolled hypertension
	Unstable angina
	Acute coronary syndrome within 6 weeks of the test
	Terminal illness
	Advanced cancer
	Psychiatric illness or dementia precluding informed consent

Abbreviations: CPET, cardiopulmonary exercise test; PAD, peripheral arterial disease.

The CPET protocol is described in detail in previous publications from our group (Loughney et al., 2014; West et al., 2015). In brief, CPET was performed according to the American Thoracic Society/American College of Chest Physicians recommendations (Fleisher et al., 2007; Palange et al., 2007). It included ECG monitoring, measurement of ventilator parameters and recording of Borg breathlessness and leg fatigue score every minute. At the end of the test, the reason for cessation was documented. The tests were blindly reported by two experienced clinicians (ND and PW). In event of disagreement, consensus was achieved between the two reporters by discussion. Other recorded variables include age, gender, height, weight, BMI, smoking status, hemoglobin concentration, ankle–brachial index (ABI) measured before and after treatment of PAD, medications and comorbidity. Resting flow‐volume loops were used to derive forced expiratory volume in 1 second and forced vital capacity. Ventilation and gas exchange variables derived by CPET included VO_2_ (absolute and weight‐adjusted), ventilatory equivalents for oxygen and carbon dioxide (VE/VO_2_; VE/VCO_2_), oxygen pulse (VO_2_/heart rate), workload and heart rate measured at LT and at peak exercise. We also recorded breathlessness and leg fatigue Borg scores at rest and peak exercise.

The first CPET was performed shortly before revascularization when the individual had been listed for the procedure. We aimed to perform the second CPET as soon as was possible after the procedure, and specifically within 3 months, though the exact timing was dependent on when the individual felt able to complete the assessments.

### 2.2 Outcome measures

The difference in VO_2_ at LT between the two CPETs was the primary outcome measure. Secondary outcome measures were VO_2_ at peak exercise and the relationship between change in VO_2_ at LT and peak exercise pre‐treatment and post‐treatment and hemodynamic measures of PAD improvement (ABI differential).

### 2.3 Sample size estimate

We assumed that a difference in VO_2_ at LT of 1 (SD 1.5) ml/kg/min would be deemed clinically significant and calculated that 26 patients would be required to demonstrate this difference, at a 5% significance level and with 90% power. We aimed to recruit 30 patients, to allow for a drop‐out rate of 10%–15% (Key et al., 2016).

### 2.4 Statistical analysis

Statistical analysis was performed using SPSS 24.0 (SPSS Inc.). Continuous variables and their differences were tested for normality with the Shapiro–Wilk test. As most were normally distributed, they were described with mean and standard deviation (SD) and compared with paired t‐tests. Differences were expressed as mean difference (md) and 95% confidence intervals (CI). Bivariate correlations were tested with the Pearson's coefficient. Statistical significance was set at 5%.

## 3 RESULTS

**FIGURE 1:**
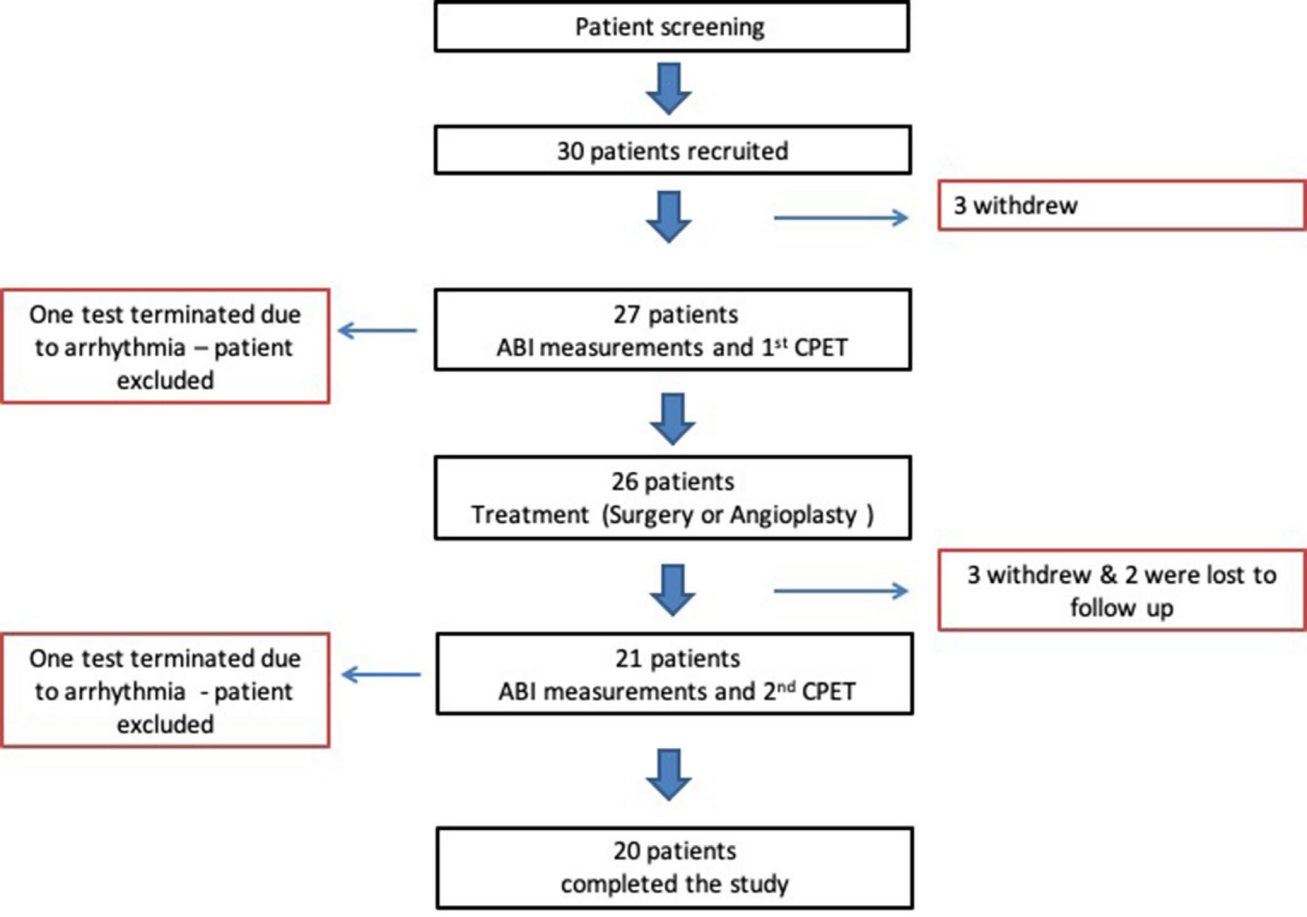
Study flowchart

Thirty patients were initially enrolled in our study; however, only 20 completed pre and post treatment CPET (Figure 1). Six patients withdrew consent, two patients were lost to follow‐up after treatment and two patients developed significant cardiac rhythm disturbance during CPET and were withdrawn by the study team. Recruitment in the study was generally slow, and, eventually, permanently halted by the COVID‐19 pandemic, which caused termination of the study before the target sample size could be achieved. Three patients developed significant leg pain during the first CPET, leading to termination. Following revascularization, two of the patients experienced no pain during the second CPET but the third subject still terminated her second CPET due to leg pain, despite normalization of her ABI (pre = 0.65, post = 0.95). One patient developed significant leg pain during his second CPET only, leading to cessation of the test; in this patient we were unable to objectively assess the hemodynamic improvement of his leg circulation due to incompressibility of the ankle arteries.

Demographics, comorbidity, drug therapy and smoking habits of the patients are given in Table 2.

**TABLE 2:** Demographics, smoking habits, and clinical characteristics

	Mean (SD) or number (%)
Age (years)	62 (8.3)
Gender M:F	15 (75%): 5 (25%)
Weight (Kg)	76.1 (11.0)
Height (cm)	167.4 (6.3)
BMI (Kg/m^2^)	27.1 (3.1)
Hb (g/L)	141.7 (11.0)
Smoking status	
Never	1 (5%)
Ex‐smoker	13 (65%)
Current	6 (30%)
Diabetes mellitus	6 (30%)
IHD	5 (25%)
Hypertension	12 (60%)
Cardiac failure	0
CKD	0
COPD	2 (10%)
Stroke (non‐disabling)	2 (10%)
Antiplatelet	20 (100%)
Statin	20 (100%)

Abbreviations: BMI, body mass index; CKD, chronic kidney disease; COPD, chronic obstructive pulmonary disease; Hb, hemoglobin; IHD, ischemic heart disease.

The revascularization procedures included ilio‐femoral bypass (four patients), common/external iliac artery angioplasty/stent (13 patients), and common femoral endarterectomy and iliac angioplasty (three patients) and were all performed without complication; no formal exercise training was provided to the patients before or after intervention. The second CPET was performed a median of 9 (2–22) weeks after revascularization.

In all but one patient, pre‐ and post‐revascularization ABI measurements were possible with an improvement from 0.51 to 0.92, hence a mean improvement of 0.40 (95% CI, 0.29–0.51, *p* < 0.0001). Sixteen patients normalized their ABI to ≥0.9

**FIGURE 2:**
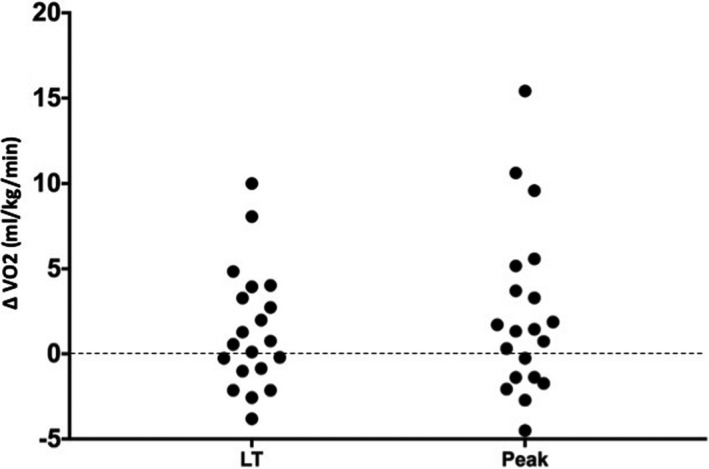
Changes in VO2 after revascularisation

CPET variables are reported in Table 3. VO_2_ improved in 12 cases at LT and in 13 at peak. Conversely, it decreased in eight cases at LT and seven at peak. Mean change in VO_2_ at LT after revascularization exceeded the value pre‐determined as clinically significant with a mean improvement of 1.43 ml/kg/min (95% CI, −0.21 to 3.08) but this difference did not achieve statistical significance (*p* = 0.085, Figure 2). However, there was a statistically significant improvement in VO_2_ at peak with a mean difference of 2.34 ml/kg/min (95% CI, 0.02–4.65, *p* = 0.048, Figure 2) as well as in Borg breathlessness and leg fatigue score, heart rate, workload and VE/VCO_2_ at peak (Table 3 and Figure 2).

**TABLE 3:** Cardiopulmonary exercise test parameters pre and post treatment

Cardiopulmonary exercise test parameters	Pre‐treatment Mean (SD)	Post‐treatment Mean (SD)	Mean difference	95% CI	*p*
FEV1 (L)	2.6 (0.57)	2.7 (0.54)	0.076	0.01–0.13	0.018
FVC (L)	3.60 (0.75)	3.68 (0.77)	0.078	−0.01 to 0.16	0.085
FEV1/FVC (%)	73.6 (5.91)	73.9 (7.22)	0.3	−2.49 to 3.00	0.825
VO2 LT (ml/kg/min)	10.95 (2.83)	12.38 (3.55)	1.43	−0.21 to 3.08	0.085
VO2 peak (ml/kg/min)	14.62 (3.96)	16.98 (5.19)	2.34	0.02–4.65	0.048
VE/VCO2 at LT	33.49 (4.44)	33.14 (4.08)	−0.34	−1.34 to 0.65	0.481
VE/VCO2 at peak	36.0 (5.11)	34.25 (4.86)	−1.79	−3.08 to −0.050	0.009
Resting heart rate (beats/min)	76 (14.67)	75 (13.02)	−1	−6.49 to 4.49	0.707
Peak heat rate (beats/min)	116 (20.0)	123 (19.0)	6	0.53–12.66	0.034
LT heart rate	94 (28)	99 (15)	4.8	−4.11 to 13.74	0.273
RER at LT	0.95 (0.07)	0.96 (0.09)	0.01	−0.02 to 0.04	0.530
RER at peak	1.14 (0.10)	1.2 (0.11)	0.05	−0.006 to 0.119	0.074
Workload at peak (Watt)	99.2 (27.6)	109.5 (29.1)	10.3	3.84–16.75	0.003

Abbreviations: FEV1, forced expiratory volume in 1 second; FVC, forced vital capacity; LT, lactate threshold; RER at LT, respiratory exchange ratio at lactate threshold; RER at peak, respiratory exchange ratio at peak exercise; VE/VCO2 at LT, ventilatory equivalent for carbon dioxide at lactate threshold; VE/VCO2 at peak, ventilatory equivalent for carbon dioxide at peak exercise; VO2 at Peak, systemic oxygen delivery at peak exercise; VO2 LT, systemic oxygen delivery at lactate threshed.

**FIGURE 3:**
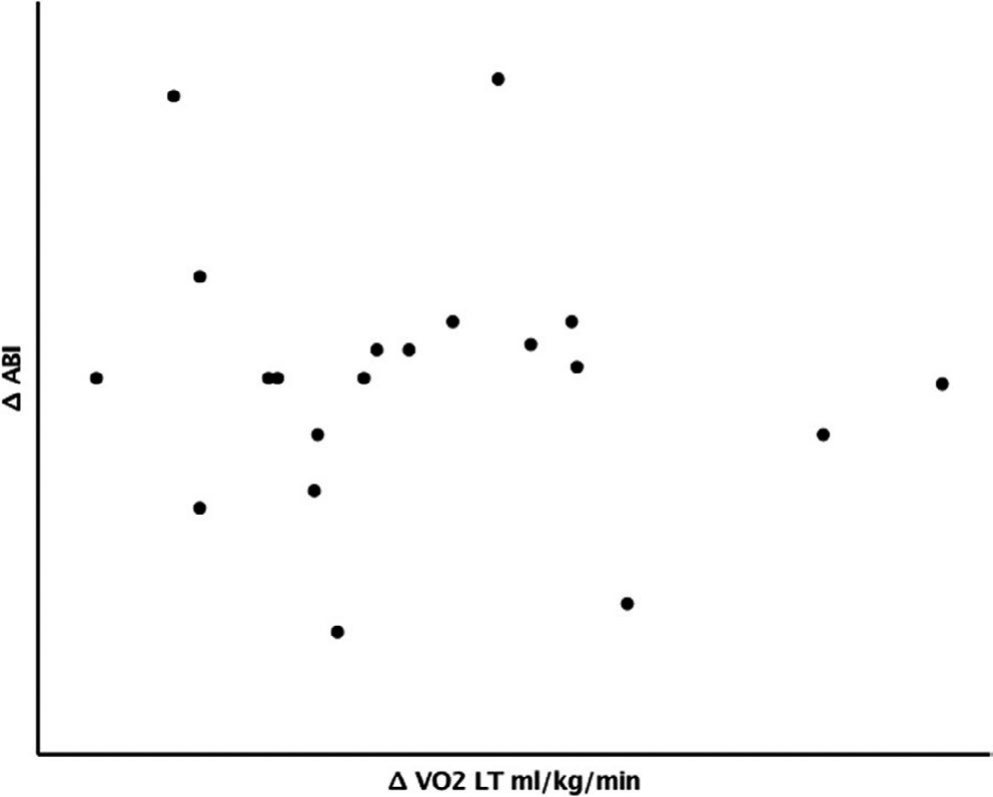
Relationship between improvement in ankle brachial index (ABI) and changes oxygen delivery (VO2) at lactate threshold (LT)

**FIGURE 4:**
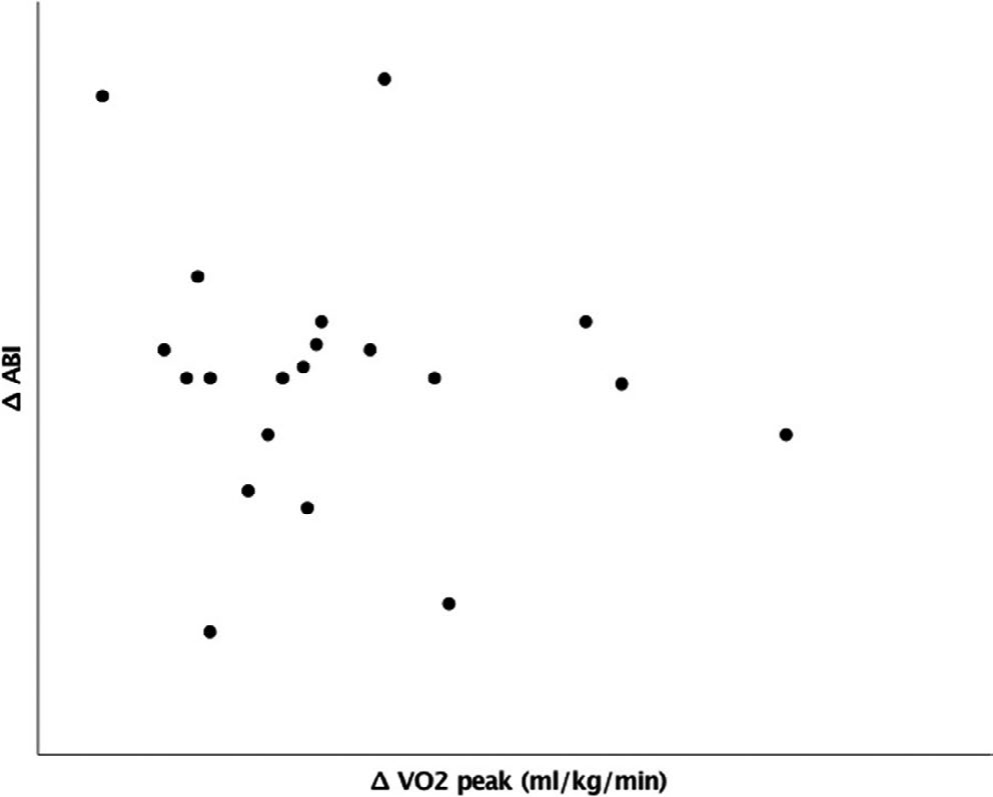
Relationship between improvement in ankle brachial index (ABI) and changes oxygen delivery (VO2) at peak exercise

There was no significant relationship between changes in VO_2_ at LT and ABI differential (*r* = −0.11, *p* = 0.65, Figure 3). Similarly, there was no relationship between changes in VO_2_ at peak and ABI differential (*r* = −0.14, *p* = 0.55, Figure 4).

## 4 DISCUSSION

Cardio‐respiratory exercise performance is limited in people with PAD; our results suggest that revascularization may improve peak ventilatory and circulatory parameters to a clinically relevant extent. Revascularization improved arterial circulation and consequently subjects exercised for longer, achieved a higher peak workload, a higher peak heart rate, a higher peak oxygen uptake and ventilatory efficiency was improved. Oxygen uptake at the onset of anaerobic metabolism improved in a majority of subjects, and exceeded our pre‐defined threshold for clinical significance, but this did not reach statistical significance (the primary hypothesis), probably because of our inability to reach our target sample size. There was no discernible link between the improvement in arterial blood supply and oxygen uptake.

During the initial (aerobic) phase of CPET, expired carbon dioxide increases linearly with VO_2_ and reflects aerobically produced carbon dioxide in the muscles. Blood lactate levels do not change substantially during this phase, since muscle lactic acid production is minimal. In the late stages of maximal exercise, anaerobic metabolism occurs because oxygen supply cannot keep up with the increasing metabolic requirements of exercising muscles. At this time, there is a significant increase in lactic acid production in the muscles and in the blood lactate concentration, a phenomenon that can be detected on CPET as a disproportionate increase in exhaled carbon dioxide (Albouaini et al., 2007). This is the LT and is a key measure of fitness in several clinical settings (Arena & Sietsema, 2011; Moran et al., 2016; O'Doherty et al., 2013; Smith et al., 2009). Oxygen delivery to the leg muscles depends on a functional cardiorespiratory system, but also on a patent arterial circulation. For this reason, one would expect early lactate rise during exercise in patients with PAD, despite adequate gas exchange in the lungs, good cardiac function and good oxygen carrying capacity by the blood and that this would improve after correction of PAD. To our knowledge, this theory has never been tested before nor has the effect of lower limb revascularization on VO_2_ been examined. We showed that VO_2_ at LT was below the level predicted and improved after revascularization but not to a statistically significant degree albeit beyond that expected and used in our power calculation, hence by a clinically significant degree.

There may be a number of reasons why a statistically significant difference was not apparent at LT whereas it was at peak exercise. First, this most likely relates to statistical power and a type 2 statistical error; our rate of drop out was double that expected plus two further subjects could not complete one or other CPET due to cardiac events during testing. Recruitment issues then ensued, most recently due to the COVID19 pandemic, precluding all but urgent or emergency surgery in our hospitals. Second, this may relate to the timing of the second test which, on average, was performed only a couple of months after the revascularization procedure, as more time may have been required for the effect of improved arterial supply to translate into improved muscle mitochondrial function. We attempted to perform the second test as soon as possible after revascularization but the interval varied considerably between subjects according to whether the individual felt able to repeat the test. Third, we have no information about activity during this period and it may be that an exercise program would have accelerated any improvement. This may be worthy of further study.

There was no correlation between changes in VO_2_ at LT or at peak exercise and ABI improvement (the secondary outcome measure). Patients with PAD have multi‐level disease and ABI is influenced by the presence of infra‐inguinal disease, which was not corrected in our cohort, so any ongoing skeletal muscle dysfunction may have impacted this relationship. We selected patients with ilio‐femoral (rather than infra‐inguinal) disease because of the greater muscle mass potentially exposed to ischemia during exercise (quadriceps, hamstrings, psoas, glutei) and excluded patients with critical ischemia because this condition (severe pain at rest with/without tissue loss) would significantly affect their ability to perform a CPET. In addition, we hoped to gain insight into the impact of occult PAD upon routine pre‐operative CPET testing, which would never be performed in an individual with critical ischemia.

The impact of PAD on lower limb muscles is multi‐faceted. PAD patients have lower limb muscles with fewer type 1 muscle fibers, fewer capillaries per muscle fiber and a reduced fiber cross‐sectional area (Askew et al., 2005). Ischemia/reperfusion injury leads to oxidative stress and impacts electron transport chain function and the production of ATP. Reduced blood flow and oxygen delivery leads to mitochondrial dysfunction possibly due to oxidative stress. Our work does not include muscle biopsies or non‐invasive assessment of mitochondrial function but we have shown that the impact of revascularization is apparent within a few weeks without use of a specific training program.

Previous studies in this area have assessed exercise capacity using a 6‐minute walking test (6 MWT) and maximal incremental treadmill CPET (Poersch et al., 2013; Pollentier et al., 2010). The 6 MWT is a self‐paced field test, does not measure maximum exercise capacity and does not allow assessment of our core outcome measures. Cycle ergometer based exercise tends to produce a lower peak VO_2_, usually 10%–20% less than treadmill exercise (Lockwood et al., 1997; Williford et al., 1994). This is due to utilization of smaller amount of muscle mass, as weight is off‐loaded during cycle ergometry (Lockwood et al., 1997), and also to the lower maximum cardiac output and lower arteriovenous oxygen differences as compared with treadmill exercise (Miyamura & Honda, 1972). However, cycle ergometry is more widely used for clinical testing and better for subjects with gait or balance instability, orthopedic limitation or obesity. It could be argued that treadmill would have been a better, more sensitive, exercise method for claudicants, as exercise by cycle ergometry does not reproduce the muscular recruitment during walking, the activity triggering symptoms in patients with PAD. However, there is a consistent relationship between aerobic capacity determined with a treadmill and cycle ergometry, with untrained subjects usually terminating cycle exercise because of quadriceps fatigue at a work rate 10%–20% below their treadmill peak VO_2_ (Arena et al., 2007; Miyamura & Honda, 1972). In fact, peak VO_2_ in our subjects exceeded a previous group of PAD patients subjected to treadmill testing (Lindner et al., 2008).

Two previous studies (Júnior et al., 2015; Tuner et al., 2008) evaluated the cardiopulmonary responses to treadmill and cycle ergometry exercise in patients with PAD. Tuner et al (Tuner et al., 2008) studied both methods of exercise assessment in 10 patients with PAD, revealing high reproducibility of the cardiopulmonary exercise responses such as the LT, peak heart, and peak VO_2_. Júnior et al Júnior et al., 2015) investigated the differences in cardiopulmonary responses and tissue saturation of calf measured by near‐infrared spectroscopy between treadmill and cycle ergometry in nine men with PAD. There was no significant difference in peak VO_2_; however, calf demand for blood in incremental exercise is higher in treadmill than in cycle ergometer, despite similar cardiopulmonary responses.

Beyond the failure to achieve the target sample size, our study had certain limitations some of which have already been discussed. Our power calculation was based on the assumption that that a difference in VO_2_ at LT of 1 ml/kg/min is clinically significant. This threshold is arbitrary, and other authors may consider it too conservative. The drop‐out rate from the study (33%) was much higher than our initial prediction (10%–15%) and a larger study may demonstrate a significant effect of PAD on the VO_2_ at LT. Further, ABI is only a crude measurement of the effect of treatment of PAD, not solely dependent on the presence of PAD, and it is influenced by infra‐inguinal disease and presence of collateral circulation. Cycle ergometry produces lower work rate and lower VO_2_ than treadmill and this might affected the sensitivity of the study. It is possible that the improvement in CPET performance could have been secondary, at least in part, to a degree of “learning” by the patients, although CPET (VO_2_ at peak in particular) has been shown to be highly reproducible in patients with cardiorespiratory disease (Barron et al., 2014). Finally, it is notable that VO_2_ decreased, in a significant minority of patients after revascularization, although the changes observed in this sub‐group were limited (all <4 mg/kg/min at LT) (Figure 2). These considerations suggest that our findings should not be seen as conclusive without confirmation by further research.

## 5 CONCLUSION

Revascularization of PAD may lead to improvement in peak exercise performance within a period of a few months although we were unable to demonstrate a significant improvement in VO_2_ at LT. PAD may impact pre‐operative exercise performance and should be considered in people considered at increased risk.

## DISCLOSURES

The authors report no conflicts of interest in this work.

## AUTHOR CONTRIBUTION

MB: patients' recruitment, measurement of ABI, data analysis and manuscript writing. AK: CPETs performance, consent and data collection. TA: wrote the protocol and facilitated patients CPET appointments. PW: CPET reporting and manuscript editing. ND: CPET reporting. JS: patient assessment, patient recruitment and measurement of ABI. FT: conception and design of the study, patients' recruitment and manuscript writing. All authors reviewed and approved the manuscript.

## References

[phy214815-bib-0001] Albouaini, K. , Egred, M. , & Alahmar, A. (2007). Cardiopulmonary exercise testing and its application. Heart, 93, 1285–1292. 17890705 10.1136/hrt.2007.121558 PMC2000933

[phy214815-bib-0002] Arena, R. , Myers, J. , Williams, M. A. , Gulati, M. , Kligfield, P. , Balady, G. J. , Collins, E. , & Fletcher, G. (2007). Assessment of functional capacity in clinical and research settings: A scientific statement from the American Heart Association Committee on Exercise, Rehabilitation, and Prevention of the Council on Clinical Cardiology and the Council on Cardiovascular Nursing. Circulation, 116, 329–343. 10.1161/CIRCULATIONAHA.106.184461 17576872

[phy214815-bib-0003] Arena, R. , & Sietsema, K. E. (2011). Cardiopulmonary exercise testing in the clinical evaluation of patients with heart and lung disease. Circulation, 123, 668–680. 10.1161/CIRCULATIONAHA.109.914788 21321183

[phy214815-bib-0004] Askew, C. D. , Green, S. , Walker, P. J. , Kerr, G. K. , Green, A. A. , Williams, A. D. , & Febbraio, M. A. (2005). Skeletal muscle phenotype is associated with exercise tolerance in patients with peripheral arterial disease. Journal of Vascular Surgery, 41, 802–807. 10.1016/j.jvs.2005.01.037 15886664

[phy214815-bib-0005] Barron, A. , Dhutia, N. , Mayet, J. , Hughes, A. D. , Francis, D. P. , & Wensel, R. (2014). Test‐retest repeatability of cardiopulmonary exercise test variables in patients with cardiac or respiratory disease. European Journal of Preventive Cardiology, 21, 445–453. 10.1177/2047487313518474 24398370

[phy214815-bib-0006] Burns, P. , Gough, S. , Bradbury, A. W. (2003). Management of peripheral arterial disease in primary care. BMJ, 326, 584–588. 10.1136/bmj.326.7389.584 12637405 PMC1125476

[phy214815-bib-0007] Fleisher, L. A. , Beckman, J. A. , Brown, K. A. , Calkins, H. , Chaikof, E. L. , Fleischmann, K. E. , Freeman, W. K. , Froehlich, J. B. , Kasper, E. K. , Kersten, J. R. , Riegel, B. , Robb, J. F. , Smith, S. C. , Jacobs, A. K. , Adams, C. D. , Anderson, J. L. , Antman, E. M. , Buller, C. E. , Creager, M. A. , … Society for Vascular Surgery (2007). ACC/AHA 2007 guidelines on perioperative cardiovascular evaluation and care for noncardiac surgery: a report of the American College of Cardiology/American Heart Association Task Force on Practice Guidelines (Writing Committee to Revise the 2002 Guidelines on Perioperative Cardiovascular Evaluation for Noncardiac Surgery) developed in collaboration with the American Society of Echocardiography, American Society of Nuclear Cardiology, Heart Rhythm Society, Society of Cardiovascular Anesthesiologists, Society for Cardiovascular Angiography and Interventions, Society for Vascular Medicine and Biology, and Society for Vascular Surgery. Journal of the American College of Cardiology, 50, 159–241. 10.1016/j.jacc.2007.09.003 17950140

[phy214815-bib-0008] Hightower, C. E. , Riedel, B. J. , Feig, B. W. , Morris, G. S. , Ensor, J. E. Jr , Woodruff, V. D. , Daley‐Norman, M. D. , & Sun, X. G. (2010). A pilot study evaluating predictors of postoperative outcomes after major surgery: physiological capacity compared with ASA physical status classification. British Journal of Anaesthesia, 104, 465–471. 20190255 10.1093/bja/aeq034 PMC2837548

[phy214815-bib-0009] Júnior, J. S. , Ribeiro‐Samora, G. A. , Ferreira, D. R. , Valeriano, M. C. P. , Santos, R. F. , Britto, R. R. , & Pereira, D. A. G. (2015). 21 Cardiopulmonary and peripheral responses to treadmill and cycle ergometer incremental exercise in patients with peripheral arterial disease: a pilot study. British Journal of Sports Medicine, 49, 7. 10.1136/bjsports-2015-095576.21 23945034

[phy214815-bib-0010] Key, A. , Ali, T. , Walker, P. , Duffy, N. , Barkat, M. , Snellgrove, J. , & Torella, F. (2016). Effect of peripheral arterial disease on the onset of lactate threshold during cardiopulmonary exercise test: study protocol. British Medical Journal Open, 6, e012763. 10.1136/bmjopen-2016-012763 PMC5168619 27993904

[phy214815-bib-0011] Lindner, J. R. , Womack, L. , Barrett, E. J. , Weltman, J. , Price, W. , Harthun, N. L. , Kaul, S. , & Patrie, J. T. (2008). Limb stress‐rest perfusion imaging with contrast ultrasound for the assessment of peripheral arterial disease severity. JACC: Cardiovascular Imaging, 1, 343–350. 10.1016/j.jcmg.2008.04.001 19356447 PMC2651026

[phy214815-bib-0012] Lockwood, P. A. , Yoder, J. E. , & Deuster, P. A. (1997). Comparison and cross‐validation of cycle ergometry estimates of VO2max. Medicine and Science in Sports and Exercise, 29, 1513–1520. 10.1097/00005768-199711000-00019 9372490

[phy214815-bib-0013] Loughney, L. , West, M. , Pintus, S. , Lythgoe, D. , Clark, E. , Jack, S. , & Torella, F. (2014). Comparison of oxygen uptake during arm or leg cardiopulmonary exercise testing in vascular surgery patients and control subjects. British Journal of Anaesthesia, 112, 57–65. 10.1093/bja/aet370 24193322

[phy214815-bib-0014] Miyamura, M. , & Honda, Y. (1972). Oxygen intake and cardiac output during maximal treadmill and bicycle exercise. Journal of Applied Physiology, 32, 185–188. 10.1152/jappl.1972.32.2.-b185 5007867

[phy214815-bib-0015] Moran, J. , Wilson, F. , Guinan, E. , McCormick, P. , Hussey, J. , & Moriarty, J. (2016). Role of cardiopulmonary exercise testing as a risk‐assessment method in patients undergoing intra‐abdominal surgery: a systematic review. British Journal of Anaesthesia, 116, 177–191. 10.1093/bja/aev454 26787788

[phy214815-bib-0016] O'Doherty, A. F. , West, M. , Jack, S. , & Grocott, M. P. (2013). Preoperative aerobic exercise training in elective intra‐cavity surgery: a systematic review. British Journal of Anaesthesia, 110, 679–689. 10.1093/bja/aes514 23393151

[phy214815-bib-0017] Older, P. , Hall, A. , & Hader, R. (1999). Cardiopulmonary exercise testing as a screening test for perioperative management of major surgery in the elderly. Chest, 116, 355–362. 10.1378/chest.116.2.355 10453862

[phy214815-bib-0018] Palange, P. , Ward, S. A. , Carlsen, K. H. , Casaburi, R. , Gallagher, C. G. , Gosselink, R. , O'Donnell, D. E. , Puente‐Maestu, L. , Schols, A. M. , Singh, S. , & Whipp, B. J. ; ERS Task Force . (2007). Recommendations on the use of exercise testing in clinical practice. European Respiratory Journal, 29, 185–209. 10.1183/09031936.00046906 17197484

[phy214815-bib-0019] Poersch, K. , Berton, D. C. , Canterle, D. B. , Castilho, J. , Lopes, A. L. , Martins, J. , Oliveira, A. R. , & Teixeira, P. J. (2013). Six‐minute walk distance and work relationship with incremental treadmill cardiopulmonary exercise test in COPD. The Clinical Respiratory Journal, 7, 145–152. 10.1111/j.1752-699X.2012.00295.x 22524795

[phy214815-bib-0020] Pollentier, B. , Irons, S. L. , Benedetto, C. M. , DiBenedetto, A. M. , Loton, D. , Seyler, R. D. , Tych, M. , & Newton, R. A. (2010). Examination of the six minute walk test to determine functional capacity in people with chronic heart failure: A systematic review. Cardiopulmonary Physical Therapy Journal, 21, 13–22. 10.1097/01823246-201021010-00003 PMC2845244 20467515

[phy214815-bib-0021] Smith, T. B. , Stonell, C. , Purkayastha, S. , & Paraskeva, P. (2009). Cardiopulmonary exercise testing as a risk assessment method in non cardio‐pulmonary surgery: A systematic review. Anaesthesia, 64, 883–893. 10.1111/j.1365-2044.2009.05983 19604193

[phy214815-bib-0022] Snowden, C. P. , Prentis, J. M. , Anderson, H. L. , Roberts, D. R. , Randles, D. , Renton, M. , & Manas, D. M. (2010). Submaximal cardiopulmonary exercise testing predicts complications and hospital length of stay in patients undergoing major elective surgery. Annals of Surgery, 251, 535–541. 10.1097/SLA.0b013e3181cf811d 20134313

[phy214815-bib-0023] Tuner, S. L. , Easton, C. , Wilson, J. , Byrne, D. S. , Rogers, P. , Kilduff, L. P. , Kingsmore, D. B. , & Pitsiladis, Y. P. (2008). Cardiopulmonary responses to treadmill and cycle ergometry exercise in patients with peripheral vascular disease. Journal of Vascular Surgery, 47, 123–130. 10.1016/j.jvs.2007.09.001 18178463

[phy214815-bib-0024] West, M. A. , Parry, M. , Asher, R. , Key, A. , Walker, P. , Loughney, L. , Pintus, S. , Duffy, N. , Jack, S. , & Torella, F. (2015). The effect of beta‐blockade on objectively measured physical fitness in patients with abdominal aortic aneurysms—A blinded interventional study. British Journal of Anaesthesia, 114, 878–885. 10.1093/bja/aev026 25716221

[phy214815-bib-0025] Williford, H. N. , Sport, K. , Wang, N. , Olson, M. S. , & Blessing, D. (1994). The prediction of fitness levels of United States Air Force officers: Validation of cycle ergometry. Military Medicine, 159, 175–178. 10.1093/milmed/159.3.175 8041458

[phy214815-bib-0026] Wilson, R. J. , Davies, S. , Yates, D. , Redman, J. , & Stone, M. (2010). Impaired functional capacity is associated with all‐cause mortality after major elective intra‐abdominal surgery. British Journal of Anaesthesia, 105, 297–303. 10.1093/bja/aeq128 20573634

